# The Intimate and Everyday Geopolitics of the Russian War Against Ukraine

**DOI:** 10.1080/14650045.2023.2222936

**Published:** 2023-06-23

**Authors:** Sven Daniel Wolfe, Olena Denysenko, Dina Krichker, Olga Rebro, Maria Gunko

**Affiliations:** a Department of Geography, University of Zurich, Zurich, Switzerland; b Department of Economic and Social Geography, Taras Shevchenko National University of Kyiv, Kiiv, Ukraine; c Gender, Identity and Diversity Research Group (GENI), University of Barcelona, Barcelona, Spain; d Department of Sociology and Human Geography, University of Oslo, Oslo, Norway; e COMPAS, School of Anthropology and Museum Ethnography, University of Oxford, Oxford, United Kingdom

## Abstract

The contributions in this Forum analyse the Russian war against Ukraine from the micro perspective of everyday life, conveyed by scholars who have been impacted at a variety of personal levels. Framed within the existential threat that continues to endanger Ukrainians and Ukraine, the contributions collected here embrace the messiness of lived experience away from the grand narratives that circulate at global scales. Instead, the authors explore a variety of processes of situated bordering that fracture not just territory, but also families and individual lives. In so doing, they shine light on the people and places where geopolitics takes shape on the ground. Taken together, this collection provides a nuanced and human-scale exploration of one of the most momentous geopolitical events in recent history.

## Introduction to the Forum

### Sven Daniel Wolfe

The Russian war against Ukraine is one of the defining geopolitical moments of our time. It has already had global impacts, from engendering food and energy crises to restructuring Europe’s post-World War security architecture. The war also resonates across digital spaces, as people across the globe – not least in Ukraine and Russia – try to digest events through WhatsApp, Telegram, Twitter, TikTok, and more. The war generates a ceaseless torrent of news, views, analysis, propaganda, misinformation, and mediatised tragedy. And, most directly, the war has impacted individual lives in ways both immediate and subtle. For people touched by the war, both on the ground in Ukraine and at a distance, there is a very real sense that a border has been drawn: life before and after 24 February 2022 will never be the same.

Nowadays, it is not uncommon to talk of World War III, of national collapse, of nuclear apocalypse, of the end of civilisation itself. This war has global significance and it inspires similarly global discussion, debate, fear, and hyperbole. In contrast, this Forum concerns itself with much smaller and more intimate scales: with individual human lives, and with stories told by people and about people touched by the war, threatened by it, implicated in it and living with it.

In the midst of the war, and connected topologically to it, this Forum attempts to ground geopolitics by exploring intimate perspectives, micro moments, and everyday life. I have written before in these pages about the value of the (extra)ordinary and seemingly mundane in understanding so-called closed contexts, with specific reference to Russia, Belarus, and Ukraine (see Wolfe 2023). I contend now that this approach is all the more valuable in the context of the war, and this is the starting point for this Forum.

Our aim is to explore the impacts of the Russian war against Ukraine from the perspective of the intimate and the everyday. This approach builds on a long tradition in feminist geography that makes sense of geopolitics by exploring situated and embodied geographies on the ground (Dixon 2015; Dowler and Sharp 2001; Massaro and Williams 2013; Pain and Staeheli 2014; Pratt and Victoria 2012). In this context, the contributions here valorise micro scales and minor moments in order to shine a different light on events that are traditionally understood as global (Deleuze and Guattari 1986; Katz 2017). Building on Smith’s (2020) foundational work on geopolitical conflict, territorial politics, and intimate spaces, this Forum endeavours to illuminate how individual bodies and lives become the sites on which territorial battles are fought – beyond the obvious daily instances of violence and atrocity. This is our understanding of the conceptual purchase of intimate geopolitics.

It can be slow and messy work, trying to make sense of the war by focusing on situated and volatile micro moments, and it often entails making oneself vulnerable in very real ways. Each of the authors in this collection has made themselves vulnerable. There is sometimes an understandable reluctance to share one’s experiences, or a sense that pursuing academic work in these circumstances is indulgent and unnecessary. There is sometimes the inability to focus due to the stupefying heaviness of the knowledge of the war. There is the fear for loved ones at risk. For those writing from Ukraine, there is another air raid siren, or another power outage, to say nothing of the personal danger or the mental exhaustion. For those with Russian passports, writing honestly means the threat of persecution and arrest, and the possibility of never returning home, alongside the guilt of being associated with these atrocities. For me personally, this year has been paralysing. I have relatives and friends in both countries.

To be clear: this Forum is not meant to take the spotlight from the existential threats that continue to endanger Ukraine and Ukrainians. At every level, from the highest echelons of Russian government discourse down to the individual actions of the invading soldiers, this war is marked by crimes against humanity and is a transparent attempt at genocide. It is created and prosecuted by an imperial superpower with long and mostly-unaddressed histories of colonial domination and domestic repression, and enacted on the ground with a callous brutality that continues to shock, despite the frequency of the atrocities. It is crucial to remember that the broader Ukrainian story underscores everything here.

Framed within this context, the authors in this collection use the intimate and the everyday to explore the intimate geopolitics of bordering. This is one of the central themes of this collection. The authors valorise the micro and the private, and in so doing, they place this perspective on equal ontological footing with the giant headlines from the world’s leading news outlets.

Olena Denysenko opens the collection with a series of intimate life stories of people who fled from Luhansk, Donetsk, Mariupol, and Kherson. She notes that the war truly began in 2014 and that 2022 represents a full-scale expansion of this long-standing aggression and occupation. In this context, she recounts the intimate experiences of her interlocutors as they were forced to leave their homes, either at the beginning of the war or during this more recent and increasingly terrible phase. In examining the confusion, fear, pain, and loss involved in fleeing the occupied territories, Denysenko builds on literature that interrogates how bodies are implicated in the construction of border and territory-making (Smith, Swanson, and Gökarıksel 2016). She cannot call the division between occupied and unoccupied Ukraine a border, but nevertheless this ‘border’ has real consequences for the lives of the people in these territories. In tracing personal stories from both 2014 and 2022, she discovers patterns between the trajectories of different people who have been affected by the war in intimate and tragic ways. In so doing, Denysenko explores how bodies and families are implicated in the ‘borders’ or fault-lines that are drawn and contested not just in physical space, but also in between families and in individual lives.

Picking up on the other side of this mass movement, Dina Krichker writes self-experientially about Ukrainian refugees arriving to a small town in Catalonia as a volunteer organiser for refugee support. At the outset of the war, local residents were active and successful in organising supplies to send to Ukraine, and they welcomed refugees with enthusiasm at both personal and institutional levels. This energy waned within the year, however, and local volunteer groups faced increasing challenges in resourcing support. Krichker unpacks these uncomfortable developments through the notions of vicarious trauma and compassion fatigue and explores the gaps between media presentations of Ukrainian refugees and their actual presence in town. To make sense of the diminishing desire for helping refugees, she turns to her work on borderscapes previously in Geopolitics (Krichker 2021), and explores how different types of divisions are constructed based on real-life interactions versus mediatised or distanced presentations of reality.

Zooming into the micro, Olga Rebro shares an auto-ethnographic intervention of her own forced double migration, illuminating what happens when a space as private and intimate as one’s home becomes imbricated in a geopolitical landscape of war. She first fled Donetsk in 2014 and settled in Mariupol, where she and her growing family participated actively in municipal politics. This political visibility made them especially vulnerable when Russian soldiers attacked in 2022. She and her family escaped from besieged Mariupol and travelled safely to Oslo, but they dream of returning and rebuilding. Rebro frames her story within the multiple modes of intimacy-politics (Pain and Staeheli 2014), highlighting the forced physical movement across space, travelling from a place of lethal danger to a foreign-but-comfortable place of safety. In this movement, she explores the inseparability of emotion from (geo)politics, and places herself and her family – their spatial relations, interactions, and practices – squarely in the spotlight of the academic gaze. In telling her stories, Rebro not only reveals the grim pragmatic realities of uprooting your life in order to survive, but also explores the intimate sensations of loss and longing that undergird the military actions that make global headlines.

Finally, Maria Gunko concludes the contributions with a report from a town in rural Armenia, situating her work in broader post-Soviet and post-socialist legacies. This framing offers a different and more subtle exploration of some of the impacts of the war. In this town, an abandoned Soviet-era factory is being inhabited and restored by a small alternative community of people fleeing the violence of the war in Ukraine and the oppression of tyrannical governments in Russia and Belarus. Contextualised within Armenia’s own turbulent post-Soviet history, Gunko dives into the micropolitics of this multinational attempt to build a kind of improvised refuge out of the shell of the Soviet built environment. Visible here is a tremulous post-Soviet solidarity, transcending national categories through the recognition of particular traumas that are common to one another, and brought into focus through the microlevel practices of working side-by-side to overcome material hardship.

Taken together, the goal of this Forum in exploring the micro and the everyday is to acknowledge and unpack the complexities and ambiguities of this war away from the narratives that function at global scales. We argue that this is especially important now, when war news is delivered instantly and non-stop around the world. Mediatised representations of war are often flattened and deprived of nuance, logically in service of state interests (Bordelon 2022; Maltby 2016). This is clear from even a casual observation of Russian state discourse since 2013 and certainly since the launch of the full-scale war. But it is also true of Ukrainian state narratives, even if there are clear, justifiable, and existential reasons for them. We think that if the academic project in human and political geography is to contribute something valuable to geopolitical debate, then it should make space for nuance and complexity – indeed, for the messiness of actually-lived humanity – in places where it has been reduced to a ‘warzone’ or rendered invisible. As presented by these authors, our hope in convening these explorations of the intimate and everyday geopolitics of the Russian war in Ukraine is to do just that.

## Exploring the “Borders” Between Occupied and Unoccupied Ukraine

### Olena Denysenko

You can never compare the experience you perceive through mass media, social networks or even personal stories to your own experience. It is absolutely incomparable … When talking to people who were not affected by the occupation, thank goodness, you understand that they JUST CANNOT comprehend, just as we did not comprehend back in 2014.

This is how a woman starts her story, a typical one for several million Ukrainian families. It is a story about experiencing war in 2022, about life under occupation and after. It is about fear and pain, difficult decisions, and loss.

Serhiy Zhadan, laureate of the 2022 Hannah Arendt Prize, emphasised the importance of speaking on behalf of the witnesses of war. This resonates with the approaches of intimate geopolitics (Barabantseva, Ní Mhurchú, and Spike Peterson 2021; Jellis and Gerlach 2017; Oswin and Olund 2010), employed here as a framework to understand the social, spatial and geopolitical processes caused by the Russian war in Ukraine. This essay focuses on intimate and ordinary lives to show how the interplay between the local and the global is manifested in establishing temporary borders both as physical barriers and as spatial fault-lines (Pain and Staeheli 2014; Pratt and Victoria 2012; Williams and Massaro 2013).

Cities in the southern and eastern regions of Ukraine were traditionally under the active influence of Russian media, both before and after 2014. This media influence contributed to their construction as geopolitical fault-line cities, where ‘the main disputes are about geopolitical alignment, foreign policy, and overall character of government’ (Gentile 2019). This situation has changed dramatically since the invasion in February 2022, however. Now, in 2023, 93% of Ukrainians believe in Ukraine’s victory in the war, including 83.5% of the Eastern region residents, 91% of the Southern residents and 96% of the Western and Central regions residents.^1^ This reflects a significant consolidation around the issues of national security, war, and the future of Ukraine. Therefore, in this paper I explore how these geopolitical fault-lines have shifted towards the ‘borders’ with the temporarily occupied territories of Ukraine, which continue to be under severe propaganda pressure, especially since 2014.

By examining the voices and experiences of families affected by the war, this essay attempts to show what spatial fault-lines emerged with the beginning of the Russian occupation of Ukrainian territory, how these temporary borders are manifested emotionally, socially and politically, and what risks are constituted for future reintegration. By doing so, this essay addresses issues of geopolitical fault-lines (Gentile 2019), bordering and border-making practices (Johnson et al. 2011; Parker and Vaughan-Williams 2009; Smith, Swanson, and Gökarıksel 2016) and border temporalities (Little 2015; Pfoser 2022). This intervention investigates these ‘borders’, their appearance and impact on consciousness and life, their political and security processes, and reveals their intimate and geopolitical meaning.

This work is based on the personal stories of people who were forced to leave their homes after the beginning of the war in 2014 and move from Luhansk or Donetsk, as well as those who left their homes in Mariupol and Kherson in 2022. It aims to reflect their experiences and their understanding of the new borders that appeared, that have divided families and separated them from their homes and their peaceful lives. This is a discussion about fault-lines, both emotional and geopolitical, informational and social, about borders on the ground that build social walls, and that shape two completely different worlds in one country in the centre of Europe.

There are parallels between the families who were forced to leave Luhansk and Donetsk regions in 2014, and those who left Kherson and Donetsk regions in 2022. People describe their feelings very similarly, as shock, confusion and rejection of reality. The state of shock often was followed by the hope that ‘everything will be resolved in a few weeks’. This is a typical picture of their initial emotions and actions:

*There were lots of people, huge crowds, buying things. I can’t withdraw money from the ATM, all stores are closed, I don’t understand what is happening … My husband comes home, gives me tickets and says: you are going to my parents. In two weeks, everything will pass, all will be well, you will come back and we will live [here]. (Poltava, 2022 about Luhansk 2014)*

Alongside confusion, and amplified by the failure of basic infrastructure and provisioning, people felt shock and fear:

*Well, to be honest, I felt the horror of what is happening when I went to Luhansk to pick up things in August-September [2014]. When we entered Luhansk, I saw destroyed buildings, unexploded missiles in the windows, 9th and 8th floors that were not there anymore. A neighbour said ‘If you’re leaving, can I take your boots or whatever grains you have left?’ That’s when it hit me hard. That’s when I realized what it was, no electricity, no water, the elevator wasn’t working. It’s a catastrophe, a real catastrophe! (Poltava, 2022 about Luhansk 2014)*

Shock and fear were also be accompanied by disbelief:

*I didn’t believe [that there would be a war]. Moreover, If I could go back, I still wouldn’t believe it. Even the first weeks in Kherson, when it was occupied, I could not believe that it was true. Perhaps because it happened so fast, but I know for sure that I didn’t expect this. Therefore, we didn’t prepare, even in terms of food or medicine, not to mention moving out. And then later we stayed until the end, when the threat of physical violence became a reality. (Ivano-Frankivsk, 2022 about Kherson 2022)*

The awareness of what was happening, and that it will last a long time, was difficult not only emotionally but also from a larger perspective: accepting that this is a war, that it is an occupation, that it is for a long time and that you need to make a decision. The decision to leave or stay, realising that the cost of this choice is your life and safety, your personal freedom, and the right to decide your own future. Fear, uncertainty, the need to make difficult decisions and all of this psychological pressure, these were the feelings of people for months.

On the other side of the scale was home, with friends and relatives who could not or would not leave. There was the former life with painfully familiar streets and destroyed buildings. To break away and to leave – for many, this meant abandonment and betrayal. They often did not dare to do this until the last moment, when the threat to life under occupation became obvious. Fleeing meant survival. People who were forced to make this choice describe it this way:

*Fear for myself, my husband and my child made us leave in the summer. By then the pressure had grown, my colleague was kidnapped and kept for several days with a bag on his head and interrogated. I was also pressured to cooperate. The awareness that someday they will come for me or my husband, and our child might be left helpless, forced us to make the decision to leave … (Chernivtsi, 2022 about Kherson 2022)*

For these people, leaving was unthinkable until the reality of the threat made it impossible to stay. At the same time, they describe the aftermath of leaving and the pain of separation:

*The hardest thing is the need to break away from my family, from my home, to leave my family members that are unprotected, knowing that you see them perhaps for the last time; this is still the most difficult moment. We were leaving and we saw the beginning of economic occupation, imposed and extremely aggressive, and we understood that it could last indefinitely long, and therefore it was hard for us to take this step, because I don’t know whether we will see them again someday … This is the hardest moment so far. And it still hurts, it does not pass, and it does not get any easier. (Ivano-Frankivsk, 2022 about Kherson 2022)*

In 2022, the rapid occupation of the Kherson region, and the south of Zaporizhia and Donetsk regions, was accompanied by the formation of occupation administrations. This led to their separation from the rest of Ukraine, guarded by front lines, checkpoints and filtration camps. Later, economic expansion and various forms of social pressure were strengthened in these territories. Physical threats, kidnappings and persecutions were designed to form a completely different social, political and informational landscape, all under the slogans of ‘denazification’.

The concept of the Russian occupation envisaged a change in the linguistic, informational and overall social landscape of these territories: all national symbols, including the Ukrainian language, were repressed by all means. Russian broadcasting with its propagandistic narratives about the war and occupation became uncontested. The widest range of methods was aimed at the artificial creation and deepening of fault-lines within Ukrainian territory, the strengthening of new ‘borders’, and their political and geopolitical reinforcement through time-tested narratives and myths (Kazanskyi and Vorotyntseva 2020) and ‘faked separatism’ (Vikhrov 2018). This mythologising was aimed at the emotional and social separation of the occupied territories in people’s minds.

The occupied territories are well described as ‘grey zones’ and ‘territories of injustice’: huge areas transformed into dangerous spaces with prosecutions and disappearances, tortures, and killings, first in 2014 and then further in 2022 (Aseev 2021; Humeniuk 2021).^2^ Two stories from refugees confirm this lawlessness:

*From the first days of March [2022], Kherson was crowded with Russian troops. There were cases when Russian soldiers simply came to an apartment, removed the door, took people away and did what they wanted. So of course, my biggest fear was for my daughter, who was studying remotely and alone at home. This is why I got her out in April. (Ivano-Frankivsk, 2022 about Kherson 2022)*

*My friend called me from the occupied territory of Luhansk region [in 2022] and says that they paid 350 euros to evacuate their daughter. It’s horrible! No one would believe that you have to pay 350 euros to get out, to avoid being shot! (Poltava, 2022 about Luhansk 2022)*

The ‘border’ with the occupied territories of Ukraine is a border of normality in political, security, economic and social senses. Many interviews show how social boundaries shifted in people who were under occupation and later fled, often at risk of physical violence or death. Once out of the occupied territories, for a long time they could not get used to the fact that ‘you can just go to the store’, or that ‘you don’t need to clean your phone all the time in fear of being checked’.

Since 2014 and until the Covid restrictions of 2020, a huge number of people regularly crossed the demarcation line with the temporarily-occupied territories, passing through formalised entry-exit control points. According to the State Border Guard Service of Ukraine, 16.5 million crossings were registered in 2019.^3^ Most of those who crossed the checkpoints in the Donetsk and Luhansk regions came to unoccupied territory for everyday reasons: social payments, buying food, administrative services. The significant number of crossings proves the close connections with other regions of Ukraine, while at the same time demonstrates the role of the demarcation line in the everyday life of people in the occupied territories.

Examining these borders through the lens of social practices (Parker and Vaughan-Williams 2009) reveals how the occupation influenced everyday life, giving rise to deep differences between the occupied and unoccupied territories. For those under occupation, the temporary border became a part of established everydayness, which normalises the demarcation line. This is not geopolitical for them, but daily reality, where crossing checkpoints was a normal social practice. Crossing the line requires special documents, waiting, being checked, and then doing the same in the opposite direction. This formalised the border through perception and experience. For those in the occupied territories, the border manifested itself as a physical barrier, but for those who fled and started new lives, the border is emotional, and behind it remained their stolen life.

For the majority of those who left, the temporary border with the occupied territories is a line of inner emotional division: lost homes and lives, lost relatives and friends. This is an intimate and difficult topic for those who left both in 2014 and in 2022. They speak reluctantly and very emotionally, because communication with those who stayed for various reasons is either gone or extremely complicated. The temporary borders became powerful spatial fault-lines:

*I will not return yet even if it will be Ukraine, please God let it be Ukraine, because it is Ukraine, but even then, I will not return there for several years. I will explain. My parents stayed in Donetsk and my cousin is in the DNR [Donetsk People’s Republic], he enlisted in the DNR military and has already been wounded a few times. And when my mom mentioned that she wished I would come so she could see her granddaughter, he said ‘Do you really think we would let her in?’ (Poltava, 2022 about Donetsk)*

This family conversation illustrates the role of the ‘border’ with the temporarily-occupied territories as many barriers at once: a physical and geopolitical barrier, a border that runs within the territory and within the family.

2014 was a turning point in the perception of these many borders (see Zhurzhenko 2021), but a particularly significant shift occurred after the full-scale invasion in 2022. Whole societies, regardless of regional identity or family ties in Russia, began to perceive the state border through the lens of security and national identity. If we consider borders ‘as a part of wider production and reproduction of territoriality’ (Johnson et al. 2011, 62), Ukraine is experiencing a period of change regarding the awareness and re-evaluation of borders and their symbolism and institutionalisation. The war completely changed for Ukrainians the awareness and perception of borders as a space of self-identification, which for centuries has experienced invasions, attacks and the pressures of colonisation. As related in the interviews, these processes caused a re-evaluation and production of a new sense of *home*, as a space of intimacy and safety that was stolen by the war as new borders were created. This forced many to locate *home* within state borders, thereby connecting intimate, national and geopolitical scales in a new social consciousness.

War has many faces, terrible, monstrous and frightening, which here in Ukraine are revealed in the eyes of children and mothers, soldiers and volunteers, relatives and acquaintances. They reflect and pass through you and your consciousness. As it turns out, war is revealed differently every day and month: first through shock, fear, and then acceptance. Later, through actions, pain and losses, and even later, through realisation and reconsideration of what is happening. In addition to pain and loss, war also opens a long path to something else: a new awareness and understanding of these events and of their historical significance. It brings a new comprehension of spatiality and borders along cultural, informational and security lines. A reinvention of one’s identity. In line with this, my essay is an attempt to discuss spatiality and borders before and after February 24, 2022. For me, as a geographer, these cannot be imagined now without reconsidering the Soviet politics and production of space. Their ideas and tools and goals had serious consequences for Ukrainian cities, industrialising and militarising them, including those in Donbas. At first Ukrainian identity and memory was erased, and now the cities themselves are wiped away.

## Compassion Fatigue as a Bordering Practice: Ukrainian Refugees in Vilanova I la Geltrú

### Dina Krichker

In the morning, on February 24th, the first thing I read when I picked up my phone was that Russia started a full-scale war on Ukraine. This news was shared by the WhatsApp chats for Russian-speaking women in Vilanova i la Geltrú, and a group of women decided to gather in front of the town hall to express their condemnation of the Russian government’s actions. We held broadsheets with anti-war slogans in Catalan and Spanish. The local news crew arrived to the town hall square, and after lunch, the residents of Vilanova found out that there is an anti-war collective in their town.

Here on, the social dynamics related to anti-war and humanitarian initiatives varied. Enthusiasm in spring 2022 was followed by scepticism and demotivation by autumn. How and why did this happen? In this intervention, I provide a short summary of humanitarian initiatives in Vilanova i la Geltrú, a small Catalan town, suggest possible explanations for the wane of the humanitarian zeal, and employ the borderscapes concept (dell’Agnese and Amilhat Szary 2015) to highlight the friction that exists between the refugees and the locals.

Before embarking on this discussion, I believe it is crucial to write a few sentences about my positionality and my own intimate association with Vilanova i la Geltrú, where I am one of the coordinators of the refugee volunteers´ group. I started this volunteering initiative because it was unbearable for me to witness silently the atrocities committed by Russia in Ukraine. I have been living in Vilanova i la Geltrú for over two years, and all along I have been the administrator of various WhatsApp groups for women in Russian. These groups proved an invaluable resource for sharing and gathering information and for organising the volunteering activities. Our volunteers’ chat on WhatsApp emerged from the Russian-speaking women’s WhatsApp group and now has over 200 participants. During my work with refugees here, I cannot recall being asked where I was from, however I have never hidden that I am Russian. A few weeks back, one of the refugees shared on her Instagram the gratitude to ‘our Ukrainian volunteer Dina’ for organising Catalan classes for children. Apparently, some refugees assume that I am from Ukraine. I don’t always feel the need or have the opportunity to clarify this confusion.

On February 28, we gathered at the town hall square yet again. This time we brought large plastic containers for gathering humanitarian aid. Our initiative was a response to a call by the Ukrainian embassy in Spain – vital medicine, food supplies and first-aid kits were needed in the war-affected areas. This is when the town administration first got in contact with the volunteers.

In a few days, the volunteers’ group together with the town administration started a big campaign for gathering humanitarian aid in all the cultural centres of the town. In one week, we gathered 32 trucks of non-perishable food items, first-aid kits and hygiene products, and delivered them to the Ukrainian embassy in Barcelona. We felt hugely supported by the town administration and the residents. The amount of the aid gathered exceeded our expectations.

This was also the time when the first refugees started to arrive. It became evident that the volunteers and the town hall complemented one another in providing first response. There were some tasks that could not be executed by the town hall. For example, there were many families in the town who stated their interest in hosting Ukrainian refugees in Vilanova. The town hall started working on a web database to connect families with refugees, but the initiative had to be abandoned. The town administration had no resources for running background checks to ensure that the refugees would be safe with the host families. Therefore, informal networks proved helpful in solving the accommodation issues and finding local hosts. The town hall referred the refugees to the volunteers’ collective on their first visit, and once in contact with the volunteers, the refugees could receive assistance.

Volunteers set four main objectives in their activity: provision of clothing and food items for the newcomers, language training for the adults, translation and interpretation services and child care. Once in Spain, the refugees would be able to access social services, including free education, healthcare and language courses. However, the limitations of the public system in Spain are widely known. One would have to wait for months before getting a response from a public institution. Therefore, the volunteers’ assistance immediately available to all the refugees coming to Vilanova was appreciated. This immediate help became possible thanks to participation from the town hall. The administration provided necessary spaces in the cultural centres and did not ask the volunteers for the paperwork usually needed when such activities are held.

The volunteers managed to make use of the enthusiasm present among Vilanova’s residents in the first months of the war. Essentially anyone willing to help could contribute in a variety of ways: organising workshops with children, participating in language training, donating clothes, toys or furniture. The fact that so many people got involved in the volunteering activities attracted the attention of businesses. Local shops and brands wanted to collaborate with our collective and offer help to the refugees. This is how the refugees got access to new clothing and personal care items, and how children got access to free English courses, volleyball and surfing schools.

If we compare the experience of volunteering in spring 2022 and autumn 2022, we notice striking differences. Both the town administration and informal support networks became less efficient and more selective in their choice of initiatives they wanted to support. Here are some examples. In spring, the volunteers could obtain a pair of glasses from a local optics store for a refugee child in a couple of hours, but by autumn, none of the local optics stores wanted to support the identical call for help. In spring, the volunteers could organise a meeting with the town administration in only a few days, but in autumn we usually had to wait for weeks in order to meet a person from the administration. In spring, we gathered 32 trucks of humanitarian aid, but in autumn we could not fill even one truck. Businesses, administration and locals became less receptive to the volunteers´ calls for help and less collaborative.

### Why does this happen?

To my understanding, and as my co-authors here have also pointed out, this war has a tendency of creating borders not only in physical space, but also in mental space. Even though there are many Ukrainian refugees residing in Vilanova now, the information about their tragedies predominantly comes from media sources, as opposed to people taking an interest in or having willingness for actual real-life interaction with them. As Victoria, a psychologist from Ukraine working with the refugees in Vilanova, put it: ‘It is not easy for our people to open up and share their grief. There are cultural reasons for this’. Apart from that, the majority of Ukrainian refugees in Vilanova do not speak Spanish or Catalan, which hinders their social integration.

This condition produces (or results from) the borderscape, a condition of being ‘in between’ on multiple levels, including social, physical and metaphorical, — the refugees have to deal with their ‘difference marked by international boundary’ (dell’Agnese and Amilhat Szary 2015, 5) on multiple levels: in public imaginations, in their everyday experiences and in their spatial practices (Krichker 2021). However, it was their very ‘otherness’ that inspired a massive response among the residents of Vilanova i la Geltrú in spring. Why are the borders now closed again?

Negative changes that happen to people who care about those who have been hurt, and who feel committed or responsible to help them, is called vicarious trauma (Pearlman and McKay 2008) or compassion fatigue (International Organization for Migration n.d..). This phenomenon is characterised by alterations in attitude – the helpers become cynical and start blaming the beneficiaries (‘Why don’t they do it themselves?’). According to Pearlman and McKay (2008, 22), vicarious trauma affects the quality of assistance provided. Therefore, if we look at the town administration’s workers and businesses that collaborated with volunteers as humanitarian agents, we can explain their change of attitude as an effect of humanitarian fatigue.

However, not only humanitarian workers suffer from compassion fatigue. Nowadays, we cannot help witnessing humanitarian crises through digital spaces. Media plays a crucial role in shaping how we feel about these crises (Moeller 1999; Tester 2001). However, experiencing strong emotions while looking at the heart-breaking images of the refugees’ crisis does not usually lead to action (Hoskins 2020). Hoskins (2020, 142) argues that active liking and sharing in digital spaces disconnects users from real world drama. The tragedies stay in digital realms and do not ignite humanitarian actions in real life.

DeFriend (2017) claims that one of the reasons for compassion fatigue lies in the gap between observing human suffering and a capacity to intervene. Surprisingly, in spring 2022, the media portrayal of the Russian war in Ukraine led to immediate actions on behalf of Vilanova’s residents and the town administration. The compassion fatigue gap became noticeable by autumn 2022, however, and nowadays, we can talk about discrepancies in the public imagination of the Ukrainian refugees – the refugees portrayed by the media and the actual refugees living in Vilanova. The borderscape profoundly divides the Ukrainians we see on the screen and the Ukrainians that live in our town.

‘I can´t leave my phone and I can´t stop reading the news. Everything happening here in Spain seems so surreal’, said Oksana from Kyiv. This makes me think about the complex multiscalar borderscape existing in Vilanova i la Geltrú: not only how the borderscape divides different groups of people, but it is also manifested within the very bodies of the refugees, creating conflicting feelings and experiences depending on specific circumstance and time. My hope is that both residents and refugees in small European towns like Vilanova will manage to navigate these complex landscapes of difference and turn the borders into points of encounter, acceptance and healing.

## What is Home? Intimate Reflections on Leaving Ukraine During the War

### Olga Rebro

We see war often through the prism of statistical reports, but a person’s life has many aspects, not only small stories in the news. I will share an autoethnographic episode when the war burst into my intimate life.

This essay is built within the framework by Pain and Staeheli (2014) where they interrogate the ways in which intimacy and geopolitics are tightly interwoven, and how these relations functions in different settings. Their focus on violence is very relevant to describing life during this war. They pay attention to the influence of wars on the manifestations of violence in intimate relations. Their work resonates with my personal experiences over the past year.

My family evacuated from Mariupol, which was engulfed in war and heavy shelling, to the safety of Norway, which became a haven for refugees.

Our story begins from a house on the shores of the Sea of Azov that was built by my husband’s grandmother when she divorced and started living from scratch. In general, almost every generation in my family has started life from scratch, several times. I saw this house for the first time in the summer of 2014, when my future husband brought me there after I was evacuated from the occupation of Donetsk. It seemed to me a paradise and we dreamed of running it as a small hotel or a bed and breakfast. The house for us was a symbol of hope and rebirth. It was like starting a new life.

We moved to Mariupol, not far away, and became politically active in the city. My husband and I were members of a political party and volunteered as assistants to the deputy of the Mariupol City Council. We wrote parliamentary requests, like trying to open a new road, or to repair some infrastructure somewhere. We were trying to solve local problems. We also started a family. I gave birth to our second child in the summer of 2021, so it was especially busy and difficult for me in the fall. This was my maternal time (Ní Mhurchú 2016), a time when it became necessary to combine the role of wife, mother, researcher, and social activist. A time for thinking cyclically about the eternal time of reproduction, reconfiguring the possibilities of political community and political identity by focusing on the mother-child subject, the born and the unborn. All this in an atmosphere of living close to the front line. When there is a lot of death around, then there is a growth of the sense of the value of life.

In 2022 the war found me in a very vulnerable position with many different stresses. I did not come to the war with emotional resources, or in a prepared state. This was due to stresses related to children, housing, and work. With two children under two years old, we spent a lot of time and energy on care. We also lived in a small apartment and the question arose: with the extra child, should we move in with parents, or rent a new apartment, or buy a house? We were in limbo and needed space to live because the family was growing. It was constant pressure. The next stress was related to work. My husband and I worked in the Donetsk State University of Management, which since 2015 had been evacuated from Donetsk to Mariupol. Suddenly it was absorbed by Mariupol State University by order of the Ministry of Education and Culture. This was a political decision. There were no explanations, no system, and no clue how to go through this reorganisation – and this was still in the context of Covid, in the fall of 2021.

My husband had a good job, but after the takeover, he was offered to quit or work in a low-paying position. So he was looking for a new job and I was transferring to Mariupol State University, working part-time at two departments. If the family is a site of geopolitics (Botterill, Hopkins, and Sanghera 2020), we can find greater meaning in my family’s search for security.

This was already a very stressful time for the family, but as we moved into winter 2021, we saw that troops were gathering at the border. A lot of journalists came to Mariupol, and we gave constant interviews to TV channels and newspapers. Ordinary people were signing up for the territorial defence. And at home we began packing this disturbing suitcase: a folder with documents, a few things for the children, candles, flashlights, chargers, snacks.

We self-evacuated as a family at the end of March 2022 from besieged Mariupol. 6 adults and two children travelled in one car – my husband and I, my husband’s mother and her husband, our two children aged 2 years and 7 months, and two other people. The road from Mariupol to Zaporizhzhia was difficult. Through the front line, mined areas, lack of fuel. In total we drove two nights, about 45 or 46 hours. We then went from Zaporizhzhia by evacuation train to Lviv, then onwards from Lviv to the Polish border, where we were picked up by a Swedish friend.

At the border there was a man attempting to get out. He was unfit for military service and had been removed from the military register in 2005. But he had his certificate renewed at the Lviv recruitment centre for trying to cross the border.

Our Swedish friend works in Norway and he drove us there by car. We had met him socially in Mariupol, before. Originally, he had offered to come get us on February 25 when the war broke out, but we refused, thinking that we would stay and defend. But Mariupol was completely destroyed and he offered to come again in March. We had no more hesitation. Different people have different opinions about Norway, but after the basements of Mariupol, we were very comfortable from the start. There was a place to sleep and no bombs were falling. At first, we lived in a refugee reception centre (Nasjonalt ankåringsenter), then with Swedish acquaintances, and then the commune where we lived on behalf of the IMDI (Directorate of Integration and Diversity) gave us a contract to rent an apartment for a period of three years. At the beginning, they paid the rent and the electricity bill for us and provided social assistance for us as unemployed people. Now, with temporary jobs, we have a salary and can pay rent and electricity ourselves. In general, life is very comfortable. The children go to kindergarten and they really like it. My husband went to an introductory program (learning the language and customs of the country), and I am working on my PhD at the University of Oslo. I will focus mainly on the spatial patterns of physical destruction caused by the Russian war in Ukraine, with particular focus on Mariupol. In my work I am raising the following issues: the spatial outcomes of the war, the patterns of physical destruction caused by the Russian war in Ukraine, social urbicide, the social and networked geographies of displaced urban communities in Ukraine, and the challenges involving physical reconstruction. I also want to look at the re-creation of sustainable livelihoods, including sustainable demographics.

Travelling from Ukraine to Norway, the journey was like a transition between worlds that are connected by the Yggdrasil tree, from Scandinavian mythology, travelling from the chthonic world to the world of humans, but with complex checkpoints. We travelled the path from the roots, from the underground world of the dead, to the upper world where there are living people, and then onward to safety. I felt this when I climbed the mountain above the fjord at the border of Sweden and Norway. The road was like a tree trunk that connects worlds.

I feel that in Norway we’re re-energising, building up the strength and resources to come back and heal and rebuild Mariupol. I think this is common. Even if people went to emigrate, even if they live abroad for ten or twenty years, still they will either return or send money for the restoration of the city.

There is great trauma from the fact that the places of your happy memories are destroyed. When we remember, for example, about childhood, about the parental home, we know that although we are far away, we could return there and feel that atmosphere of happiness again. But when your city is destroyed, you experience such complex emotions, as if your memory and history were erased. Our family home on the Sea of Azov is not a house where we can receive tourists and guests, or where we could dream about building a life or running a bed and breakfast. Right now, it is literally occupied by Russian soldiers.

The home is about the intimate and the personal. We invite relatives and friends to the home. But when there are enemies in the home, those who wish you death even when you evacuated, a piece of your soul still remains there. This is why it hurts when your home and your memories are destroyed. It is a part of you, even though you are far away.

If this is an example of one family, we can extrapolate to the millions of other Ukrainians who are in forced migration. Ukraine itself is our home, and everyone keeps it in their hearts. There are the graves of our ancestors, our memories, our houses and our things. We do not want simply to cross it out and forget it, despite everything. No, we will be there until the end, even if it will be many years later.

## De-Bordering in an Armenian Small Town “Nothingness” Against the Russia-Ukraine War

### Maria Gunko

**Figure 1.**
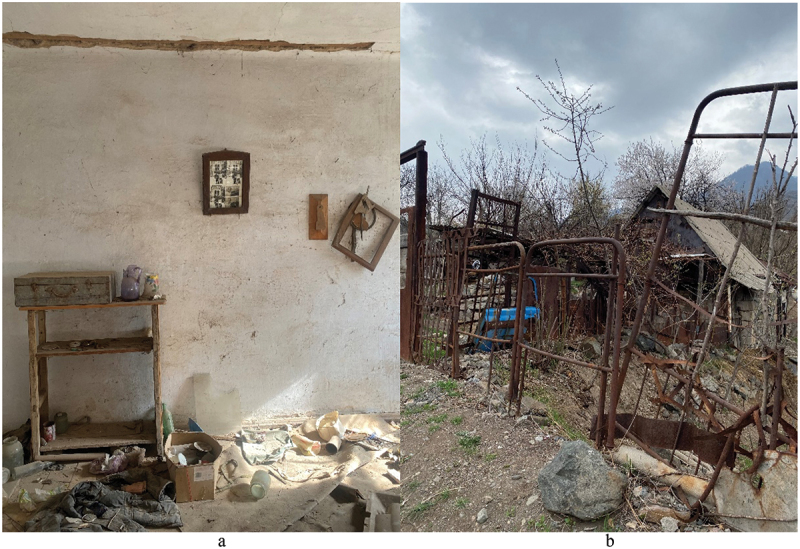
a. Abandoned house in Ajidzor. 1b. Typical fences around houses in Ajidzor made from scrap metal. Here, from old bed frames and pipes which were a part of the centralised heating system dismantled after the collapse of state socialism. Photos by Maria Gunko, March 2023.

I arrived in Ajidzor, a small town in one of the mountainous provinces of Armenia, in early August 2022.^4^ A ‘commune’ of emigrant and refugee artists from Russia, Ukraine, Belarus, and Iran was the reason this place gained my attention while I was searching for a field site to pursue my DPhil ethnographic research within the ‘Emptiness: Living Capitalism and Democracy after Postsocialism’^5^ project. These people were all fleeing state-induced violence, though the degree to which they were exposed to it was very different. Some were unwilling to put up with the politics in their respective countries. Others ran from actual prosecution, and, finally, the rest were escaping war. The condition on which they gained an opportunity to settle in Ajidzor was to facilitate the emergence of a residence for creatives in an abandoned textile factory building (hereafter, *Fabrika*), which required profound renovation based on volunteer work before it could host such a space. For a long time, it was unthinkable anything new could emerge on the ruins of Soviet modernity in a town where broken pavements, wilding land plots, dark windows in vacant apartments, derelict buildings with signs of scavenging, and fences made of rusty scrap metal seemed to distinctly signal that life has gone elsewhere (Figure 1a,b). It was almost as unthinkable as a war between Russia and Ukraine. However, this unparalleled act of military aggression sparked fast-paced transformations, contributing to ruination and desertion of some territories in the post-Soviet region, while simultaneously bringing new life in others such as in the case of Ajidzor.

After the collapse of state socialism, the countries of the former Soviet Union embarked on various paths with at times dissimilar outcomes and patterns. Among the similarities between them is a continuous ‘hollowing out’, that is, depopulation and decrease of densities especially found in small towns and rural areas – from land use to transport routes and water pressure in the pipes. This ‘hollowing out’ constitutes a new reality of which in Armenia people say ‘there is nothing here’ *aistegh vochinch chka/ban chka* [այստեղ ոչինչ չկա/բան չկա]. Yet, this phrase does not describe actual nothingness. *Vochinch chka/ban chka,* similar to other descriptors related to ‘emptiness’ found in the post-Soviet realm, is used as an explanatory emic concept for a something that was produced by the changing relations between people, space, state, and capital in the course of postsocialism (Dzenovska, 2020).

While slow violence, i.e., capital and state neglect, contributed to gradual dilapidation, wars were the most extreme manifestation of postsocialist change that shaped the devasted and ravaged landscapes (Artiukh 2022; Gunko 2022). Territorial conflicts, tensions associated with reterritorialization, and the contestation of new borders burst in the region soon after the dissolution of USSR being rooted in the history of colonial conquests and ethnic conflicts within the Russian Empire and aggravated by the Soviet nationalities policies (Grant 2016; Hirsch 2014). Some of these tensions were to different degrees pacified, but none resolved to this day (e.g. Derluguian 2005; Waal 2013). Until recently, among the bloodiest clashes in the post-Soviet space were the wars in Chechnya in 1994–1996 and 1999–2009 (Russia) and Abkhazia 1992–1993 (Georgia). Furthermore, since the late 1980s there is a continuous conflict between Armenia and Azerbaijan over Nagorno-Karabakh. To date it resulted in three open wars – the First Nagorno-Karabakh war (1991–1994), the Four-day war (2016), and the Second Nagorno-Karabakh war (2020). Moreover, during the ‘no-peace-no-war’ periods (Papazian 2008), there are occasional military clashes along the borders of the two countries and infrastructural violence against residents of Nagorno-Karabakh^6^ that reflect the broader processes of their marginalisation, abjection, and disconnection (Rodgers and O’Neill 2012).

Due to a somewhat unofficial, yet intrinsic relationship between Armenia and Nagorno-Karabakh (Panossian 2001), Armenian ‘nothingness’ (*aistegh vochinch chka/ban chka)* is by and large the result of the ongoing feud with Azerbaijan. The latter has become constitutive for shaping contemporary Armenian spatialities, the state, and everyday life (Waal 2013; Papazian 2008). It brought about the blockade of Armenia by Azerbaijan and Turkey during the first years of its independence, causing an unprecedented country-wide energy crisis of the early 1990s, known as the ‘Dark years’– *Mut tariner* [Մութ տարիներ]. Between 1992 and 1995, the country was basically deprived of heat and light. The negligible available media reports and photo chronicle of that time showcase Armenia of the early 1990s as a country of darkness, cold, silence, and shortages (Shapiro 1993). Though the electricity supply was restored by mid − 1995, Armenia never fully recovered from those events. According to various estimates around half a million people emigrated, leaving behind housing vacancies. Centralized Soviet-made infrastructures crumbled or were totally dismantled, giving way to highly individualised solutions for infrastructural provisioning and maintenance that polarised population according to wealth. Furthermore, numerous industrial enterprises stayed closed, becoming grounds to salvage value accumulation from the reuse and recycling of the remnants of Soviet modernity (Khatchadourian 2022).

Since the early 1990s the Ajidzor textile factory stayed mostly vacant, with occasional use as a fruit and vegetable warehouse. In 2019, *Fabrika* was bought by an Armenian diaspora businessman who planned to turn it into a ‘creative’ (in his words) space that could generate value with ideas varying from an IT hub to an art residence. The project gained the name *Abastan*^7^ [Ապաստան] – meaning shelter in Armenian. However, the Covid − 19 pandemic struck followed by the Second Nagorno-Karabakh war, making his vague plans even vaguer. Then Russia invaded Ukraine in February 2022.

The initial call to join *Abastan* was circulated on the internet in late 2021. However, it did not gain much attention. It was the Russia-Ukraine war and the events that followed that became the catalyst for an intense inflow of creatives to Ajidzor since early June 2022 when a new, targeted call was circulated through channels associated with the creative world, e.g., through the Artists at Risk initiative and the like. Unable to work freely and express themselves, Russians and Belarusians (as well as Iranians) were driven by the fear of hypothetical repressions, as well as fleeing actual political terror. Meanwhile, Ukrainians were seeking refuge from the full-scale offensive war fought in their homeland. Yet, upon arrival to Ajidzor, what they encountered was not a ready-made residence, but a dilapidated vacant factory:

..*.a massive building filled with garbage and remnants of factory’s former industrial activities. Standing beside it, I saw a small number of people of different ages and backgrounds. Yet, they were all united by the fear of war and state terror, and their unwillingness to put up with it. I saw people who fled and were given shelter. But to make a life here, of course, there was a huge amount of work.* (Vadim, 38 y.o.)

Looking at borders in a multi-scalar way, one can trace how they function not just as markers of space and nations, but as a fluid and blurry phenomenon that both excludes and includes, separates and brings together (Wastl-Walter 2011). If we understand them as constitutive to the ordering of societies facilitated by the processes of othering (Paasi 2011), being among ‘vulnerable others’ (Ambrosini 2022) and working together for a greater cause – that of creating a home far from home – seems to explain the process of de-bordering within *Abastan*. People of different professions, previous life experiences, and nationalities, including those of warring countries were drawn together by volunteer work side-by-side for the prospective residence and establishment of a common living space:

*At first, I met these interesting and sweet people who were doing something out of pure enthusiasm. And I wanted to contribute somehow – tear off old plaster, glue, paint. When you are scraping pigeon poop off the walls with someone or attempt to cover the roof with garbage bags and film, so rain doesn’t flow over your head, this inevitably unites and builds sort of kin ties. This dissolves borders*. (Inna, 31 y.o.)

**Figure 2.**
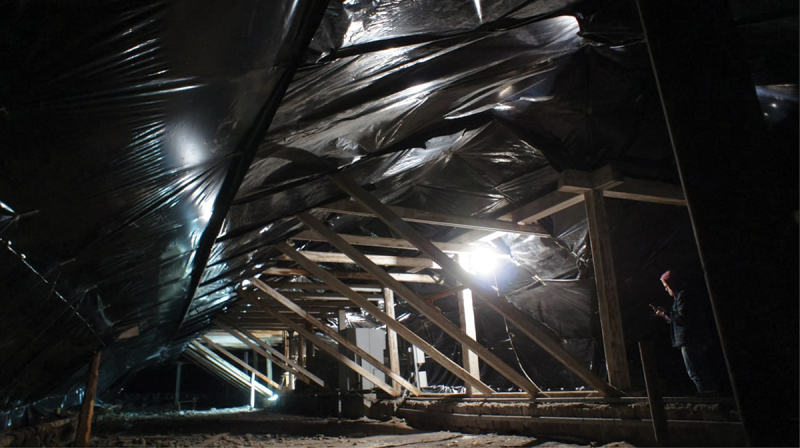
Repair works of Fabrika’s roof. Photo by Yuren, December 2022.

The desire to escape violence seemed to underlie the commonality and de-bordering that I witnessed in *Abastan*, but they crystallised through a common and embodied effort to repair the *Fabrika* (Figure 2). Following the upgrading of *Fabrika*, new meanings and activities also emerge for other abandoned buildings, such as opening a café in a former shop along with the repopulation and renovation of vacant apartments by *Abastan* members who chose to winter in Ajidzor when *Fabrika* was closed due to the leaking roof. This contributed to facilitating closer dialogue among the predominantly Slavic-speaking *Abastan* and the Armenian residents of Ajidzor.

Being a small nation stuck between powerful empires, Armenians have a long history of experiencing oppression and violent displacements (Badalyan Riegg 2020). Since the collapse of state socialism, the country constantly lives in fear of a full-scale war with Azerbaijan (Suny 1993). This provides an explanation for the sympathetic attitudes expressed by Armenians not only towards Ukrainians, but also towards the Russians and Belarusians fleeing state terror. As several of my interlocutors pointed out, upon arriving to Armenia they felt like children having to re-start and re-learn living. But in this situation, they found care and support being almost literally ‘led by hand’ by the members of the host community in Ajidzor and beyond (Georgiy, 33 y.o.). This defies the images of separation that are portrayed by the media in the Global North, where the origin of one’s passport defines whether a person is welcomed or not.

While borders divide nations on the macro level, on the micro they seem to define people much less. In winter 2022, when I began my long-term ethnographic fieldwork in Ajidzor, I found *Abastan* and the residents of Ajidzor to be in an ongoing process of rapprochement though neighbourly interactions facilitated by the winter’s hardship. The heritage of the infrastructural disruptions of the ‘Dark years’ are common to all Ajidzorians and require some collaboration in coping:

*Eventually, it became clear that it is possible and even necessary not only to help each other [within Abastan], but also to help Ajidzorians. At one point, Anton [neighbour] tells me: ‘I’m running over to Grandma Arpi, are you coming with me?’ I asked what had happened, ‘A pipe in her bathroom burst’. So, we ran to Grandma Arpi to engage in a two-day fight with an absolutely rusted water pipe. But, we did not win until our team was joined by several Armenians of different ages and professions. On the evening of the second day there were about seven of us in that cramped bathroom.* (Yuren, 50 y.o.)

Times of war constitute conditions for the abrupt cut of ties, as well as the most extreme situations that prompt individuals to demonstrate solidarity with one another (Josiassen, Kock, and Assaf 2022). Shared memories and fears of violence are the basis of solidarities in Ajidzor, reinforced by a common experience of overcoming everyday hardship. Though not without controversies, it is undoubtable that *Abastan* is literally building a new future for Ajidzor that was desolated by the slow violence of the state and devalued by capital. This materially manifests in the gradual physical upgrade of the *Fabrika*. My interlocutors describe this experience as something between a hippy-commune and a summer camp, with an implied objection against borders and bordering, be that between states, or different nationalities, genders, sexualities, or age groups. At its peak in summer 2022, over 60 people cohabitated at *Fabrika’s* premises and local hostel: men, women and non-binary people aged between 18 and 50. As of winter 2022-2023, around 20 of them remained in Ajidzor, while *Abastan* lays low before re-opening in spring.

‘I felt home, I felt welcomed and accepted’ – this is a common narrative expressed about *Abastan* and Ajidzor in general, regardless of whether the people with whom I spoke visited for several summer weeks or stayed over winter. Escaping war and terror, they paradoxically found, even if temporary, home in a country which is itself *de facto* in a state of war that throughout the years has produced various types and sites of destruction. Yet, the community that these people created gradually, both materially and metaphorically, seems to fill in to some extent the ‘nothingness’ left in Ajidzor by postsocialist reordering, the ‘Dark years’, and the ongoing conflict with Azerbaijan. As argued by Thomas Hylland Eriksen (2015), it is the small places that help us better understand large issues. They are ‘fundamental loci at which geopolitical power is made and contested’ (Williams and Massaro 2013, 752). On the background of the Russian war against Ukraine, exploring Ajidzor and *Abastan* at the microscale of human activity and social relations, their mundane interactions and changing local practices, helps to unpack the processes of warring, bordering and de-bordering that constitute and contest the macroscale geopolitical order.

## Conclusion

### Sven Daniel Wolfe

It has now been over a year since Russia’s full-scale invasion of Ukraine, and over nine since the Russian annexation of Crimea and destabilisation of the Donbas. I was living in Russia at the time of the Euromaidan protests, and when the Revolution of Dignity took place in February 2014, I was staying with my parents-in-law in Sochi to attend the Winter Olympics. Russian television – quite a bit different than what it is now – broadcast the Ukrainian skier Bogdana Matsotska’s withdrawal from the Games in order to return to Kyiv and support the Maidan. Her protest prompted a lot of discussion in our home about politics in sport. Because of the volatility, we were Skype-calling the family in Dnipro every day to make sure they were safe. On that day, we discovered they were equally divided about Matsotksa’s decision. But the whole family – in both Ukraine and Russia – understood that President Yanukovych was corrupt and blind to the concerns of ordinary people. He was a politician after all. These things were obvious and did not need to be said. It was no different in Russia and we all knew it.

At that time, Tatiana Zhurhenko had just published ‘We Used to Be One Country’ (Zhurzhenko 2013), but it would be many years before I discovered this excellent work. In it, she describes the impacts of the formation of an international border between Russia and Ukraine, and explores how this exacerbated the asymmetries in economic and social provisioning for local communities. But she also notes that ‘neither Russian nor Ukrainian citizens perceive the new border as a cultural boundary’ (Ibid., 221), and this describes perfectly my family’s experiences. Now, of course, things are different. There are extraordinarily painful cleavages in the family among cultural, political, national, and generational lines. My wife’s WhatsApp family chat is a jumble of chauvinistic Russian propaganda (forwarded many times like a ghastly chain letter), followed by carefully researched and referenced rebuttals, and then interspersed with ordinary correspondence regarding the weather, and the occasional banal forwarded picture of flowers or well-wishes for the weekend. Digital everyday geopolitics as an exercise in the surreal.

Part of my motivation in convening this Forum is to give light to these seemingly insignificant interactions. We argue here that intimate spaces and small moments are more than anecdotes to be recounted in personal conversation, or used as vignettes to facilitate entry into discussing global affairs at more ‘proper’ scales. Instead, we contend that moments like these are where geopolitics takes shape. The family I married into never needed national, cultural, political, or ethnic identities before. Since 2014 and certainly since 2022, these markers have become essential. These are questions literally of life and death. People belonging to the nation of one part of my family are murdering people in the nation of the other part of my family. No one visits for the holidays anymore. In all senses, the borders have become ultra-rigid. This is what geopolitics looks and feels like, taking shape in intimate spaces and lives.

The contributors to this Forum all have experienced these intimate geopolitics of bordering in various ways and at different degrees of intensity. They explore it here either by centring their own experiences or by exploring the war as expressed in the lives and life courses of others. Reading their contributions as a whole, I notice the resonances between their different treatment of borders, bordering, and the notion of home. For instance, Denysenko discusses how perception and experience contribute to the formation of borders, and highlights the distinction between the physical ‘border’ with the temporarily occupied territories, and the emotional border behind which lie the remains of stolen lives. Home is on the other side. In a broader sense, however, she describes the ways in which so many people – including my relatives in Ukraine – have come to locate a new sense of home within national borders, and in this light, reconsider crucial questions of identity.

For Krichker, borders are drawn by the international boundary and then maintained and reinforced by spatial practice and everyday experience. The question for Krichker is how the Ukrainian ‘otherness’ inspired heartfelt generosity at the outset of the full-scale war, only to be extinguished after less than a year. Otherness is so often a marker for discrimination rather than compassion, so I found the open-hearted reaction of the locals in Vilanova remarkable. I admit I was not surprised to learn of their compassion fatigue, however. This also raises questions of what awaits those refugees who might decide to build their home abroad. In Switzerland I have seen appalling instances of anti-Yugoslav racism directed at the now-grown children of those who fled that war. Once the blue and yellow flags of support come down from the cities of Western Europe, will Ukrainian refugees face similar attitudes? And what about Ukrainians with darker skin, and people from Africa and Asia studying or working in Ukraine? Who is allowed to call a place *home*? What about refugees from other wars? Are these people marked by an otherness that denotes a selective compassion? Are people inured to the traumas of certain other peoples?

Gunko hints at these other worlds of war, as she investigates in Armenia the micro spaces of shared experience that help transcend the borders established by violence and national or political categorisations. Coming from a shared post-Soviet experience, and working together to overcome everyday hardship, the building of a creative space in Abastan shows a quiet alternative to the narratives of division that – quite naturally, to be fair – are so present these days. It is a fragile thing and easily extinguished, but to me Abastan represents a glimpse of different and better ways of relating. It is too much to imagine that this kind of improvised community could overcome the imperialisms, colonialisms, sexisms, and other intertwined legacies of oppression that continue to suffuse these regions. It is not even accurate to say that it gives hope, but to me at least it offers something like a respite, however slight, from the ongoing horrors.

Rebro’s work also touches on the ideas of building and rebuilding, even as she speaks to the heart of our attention to a geopolitics of the intimate and the everyday. Here we see not the abstractions of theory or global scales, but the simple human experience of a young mother building her life, and then continually disrupted and displaced by war. Her desire to return home to Ukraine and rebuild resonates with Brickell’s (2012) notion that home is where geopolitics and history actually emerge. In this light, it becomes clear how the intimacy and privacy of the home becomes a geopolitical space. It also brings up personal questions with larger implications: Rebro and her family are comfortable now in Oslo, but are they home? And will her children feel the same as she does? In the meantime, I find myself thinking of her family’s holiday house on the Sea of Azov, and wondering if the Russian soldiers are still quartered there, and for how much longer.

## Notes

1. Sociological survey conducted by Razumkov Centre in February-March 2023 in the territories controlled by the Government of Ukraine. Available at: https://razumkov.org.ua/napriamky/sotsiologichni-doslidzhennia/gromadska-dumka-pro-viinu-peremogu-ta-garantii-bezpeky-liutyi-berezen−2023r

2. The list of territories temporarily occupied by Russian Federation approved in Order № 309 as of 22.12.2022 by Ministry for Reintegration of the Temporarily Occupied Territories of Ukraine. Available at: https://zakon.rada.gov.ua/laws/show/z1668–22#Text

3. Statistics provided by State Border Guard Service of Ukraine available at: https://minre.gov.ua/page/kontrolni-punkty-vyizdu-vyyizdu-kpvv-pomisyachnyy-peretyn-liniyi-rozmezhuvannya

4. Data was drawn from participant observations and interviews done in August, November, and December 2022, January – March 2023. Interlocutors and the town are pseudonymized.

5.
https://emptiness.eu

6. Since Armenia’s defeat in the Second Nagorno-Karabakh war, Azerbaijan that gained control over the surrounding territories, recurrently cuts gas and electricity supplies to Nagorno-Karabakh. In December 2022, along with numerous disruptions to gas, electricity, and internet infrastructures of Nagorno-Karabakh, Azerbaijani eco-activists backed by the country’s authorities blocked the Lachin corridor – the only remaining road via which Armenia supports and supplies Nagorno-Karabakh (Panossian 2001). As of April 2023, while finalising this text, the road has been closed for over four months. This defined a dire situation in the region with blackouts, lack of heating, food and medicine shortages (Amnesty International 2023). Some of my Armenian interlocutors compare this situation to the ‘Dark years’ in Armenia of the early 1990s pointing to the continuity of hardship experience by the nation.

7. Western Armenian pronunciation.
